# A national training program for simulation educators and technicians: evaluation strategy and outcomes

**DOI:** 10.1186/s12909-016-0548-x

**Published:** 2016-01-22

**Authors:** Debra Nestel, Margaret Bearman, Peter Brooks, Dylan Campher, Kirsty Freeman, Jennene Greenhill, Brian Jolly, Leanne Rogers, Cobie Rudd, Cyle Sprick, Beverley Sutton, Jennifer Harlim, Marcus Watson

**Affiliations:** HealthPEER, Faculty of Medicine, Nursing & Health Sciences, Monash University, Victoria, 3800 Australia; University of Melbourne, Victoria, 3010 Australia; Queensland Health Clinical Skills Development Service & University of Queensland, St Lucia, Queensland 4072 Australia; Postgraduate Medical Education Unit, WA Country Health Services, 189 Wellington St, Perth, WA 6000 Australia; Flinders University Rural Clinical School, Renmark, South Australia 5341 Australia; Faculty of Health and Medicine, University of Newcastle, University Drive, Callaghan, NSW 2308 Australia; Kirsty Freeman Consulting, Currambine, Western Australia 6028 Australia; Flinders University, Sturt Rd, Bedford Park, South Australia 5042 Australia; Health Education Australia Limited (HEAL), Level 7, 118 Queen Street, Victoria, 3000 Australia

**Keywords:** Simulation, Program evaluation, Health workforce, Faculty development

## Abstract

**Background:**

Simulation-based education (SBE) has seen a dramatic uptake in health professions education over the last decade. SBE offers learning opportunities that are difficult to access by other methods. Competent faculty is seen as key to high quality SBE. In 2011, in response to a significant national healthcare issue – the need to enhance the quality and scale of SBE - a group of Australian universities was commissioned to develop a national training program - Australian Simulation Educator and Technician Training (AusSETT) Program. This paper reports the evaluation of this large-scale initiative.

**Methods:**

The AusSETT Program adopted a *train-the-trainer* model, which offered up to three days of workshops and between four and eight hours of e-learning. The Program was offered across all professions in all states and territories. Three hundred and three participants attended workshops with 230 also completing e-learning modules. Topics included: foundational learning theory; orientation to diverse simulation modalities; briefing; and debriefing. A layered objectives-oriented evaluation strategy was adopted with multiple stakeholders (participants, external experts), methods of data collection (end of module evaluations, workshop observer reports and individual interviews) and at multiple data points (immediate and two months later). Descriptive statistics were used to analyse numerical data while textual data (written comments and transcripts of interviews) underwent content or thematic analysis.

**Results:**

For each module, between 45 and 254 participants completed evaluations. The content and educational methods were rated highly with items exceeding the pre-established standard. In written evaluations, participants identified strengths (e.g. high quality facilitation, breadth and depth of content) and areas for development (e.g. electronic portfolio, learning management system) of the Program. Interviews with participants suggested the Program had positively impacted their educational practices. Observers reported a high quality educational experience for participants with alignment of content and methods with perceived participant needs.

**Conclusions:**

The AusSETT Program is a significant and enduring learning resource. The development of a national training program to support a competent simulation workforce is feasible. The Program objectives were largely met. Although there are limitations with the study design (e.g. self-report), there are strengths such as exploring the impact two months later. The evaluation of the Program informs the next phase of the national strategy for simulation educators and technicians with respect to content and processes, strengths and areas for development.

**Electronic supplementary material:**

The online version of this article (doi:10.1186/s12909-016-0548-x) contains supplementary material, which is available to authorized users.

## Background

Educators may require unique and specific pedagogical skills to effectively support learning through simulation. As McGaghie et al. state: Simulation-based education (SBE) “is *not* easy or intuitive; clinical experience alone is *not* a proxy for simulation instructor effectiveness” [1]. As simulation is increasingly used in health professional education, there is a concomitant need for appropriately skilled teachers. While there are many faculty development programs in SBE, we could not locate any related theoretical or empirical published reports. From an international perspective, Navedo and Simon (2013) provide a descriptive account of thirteen multi-day continuing education simulation instructor courses [2]. Although it is unclear how the programs were identified (or sampled), they write, “Typically, these programs are on-site, intensive experiences with established and well-defined learning outcomes.” (p596) The authors report features common to the courses such as, “overviews in teaching and learning theory, introduction to simulation-based learning, orientation to the equipment, debriefing fundamentals, and management of common problems and offer hands-on opportunities to practice.” (p596) For simulation educators who seek further scholarly advancement, the authors describe eight award programs with key components on SBE. Zigmont et al. (2015) describe the content of basic and advanced courses for simulation educators [3] recommending Kern et al’s (1998) six steps for curriculum design [4] and mapping course content to the blueprint of the Certified Healthcare Simulation Educator standards of the Society for Simulation in Healthcare http://www.ssih.org/Certification/CHSE). Nestel et al. (2013) also offer a descriptive account but of a national strategic approach to SBE and the role of programmatic factors [5]. They argue for enabling connections of national, state and local initiatives such that programs are not seemingly *ad hoc* but coordinated and facilitated through development of communities of practice. Reviews of effective SBE cite the need for targeted training for faculty [1, 6].

In Australia, the healthcare simulation education community has experienced a period of particularly rapid growth. This is in part a response to the actions of Health Workforce Australia (HWA), a body “established to meet the future challenges of providing a health workforce that responds to the needs of the Australian community” [7]. Disestablished in late 2014, HWA was federally funded and closely linked with state and territory governments. Like most Australian bureaucracies, HWA faced the challenge of ensuring equity throughout a nation, which has a huge geographic area, but with the majority of the population concentrated in major coastal cities. Additionally, it had a broad focus, including issues such as enhancing clinical training, workforce planning analysis and supporting the role of international health professionals. Within its program of *clinical training reform* [7], HWA contributed more than $90 million over three years to enhance SBE within Australia, to develop the health workforce.

### The AusSETT Program

In 2010, HWA commissioned surveys of health professional curricula in order to establish current and potential uses of simulation. The reports, which included medical and nursing schools, identified the key issue of insufficient trained faculty to maximise the benefits of SBE [8]. Responding to this issue in 2011, HWA funded a consortium of Australian organisations, who delivered postgraduate courses in simulation, to develop a national train-the-trainer program for simulation educators and simulation technicians. The latter were considered to be a group of faculty who provided technical support in the implementation of SBE. The resulting Australian Simulation Educator and Technician Training (AusSETT) Program aimed to provide experienced simulation faculty with a curriculum and skillset to train others (see Table 1). The Program was designed to have relevance to all health professions, at all levels of training and across every state and territory in Australia.

**Table 1:** Participants’ ratings of the extent to which they met learning objectives for the modules in the AusSETT program

	Number	Mean	Median	SD	95 % Confidence interval for mean	
					Lower	Upper
C1: Simulation-based education: Contemporary issues for healthcare professions						
1 Outline common definitions of simulation-based education (SBE)	254	4.68	5.00	0.90	4.56	4.79
2 Describe various types of simulation activities	254	4.72	5.00	0.95	4.60	4.84
3 Describe the role of a ‘safe’ learning environment for SBE	254	4.65	5.00	0.95	4.53	4.77
4 Debate the benefits of teaching and learning with simulation	254	4.62	5.00	0.96	4.50	4.74
5 Debate the limitations of teaching and learning with simulation	253	4.40	4.00	0.99	4.28	4.53
6 Give examples of simulation modalities best suited for learning specific skills	253	4.31	4.00	1.00	4.19	4.44
7 Access publicly available resources on SBE	253	4.35	4.00	1.10	4.21	4.49
8 Identify professional development opportunities for simulation educators and technicians	250	4.38	4.00	1.08	4.24	4.51
9 Outline key research findings on simulation as an educational method	252	4.43	5.00	0.96	4.30	4.55
10 Outline theories that inform SBE	251	4.51	5.00	0.99	4.38	4.63
11 Explain key components of SBE, framed by available evidence	249	4.37	4.00	1.04	4.24	4.50
C2: Training simulation educators						
12 Demonstrate implementation of educational theory in teaching practice	247	4.51	5.00	1.01	4.38	4.63
13 Compare and contrast SBE methods used in health professional education	245	4.68	5.00	0.92	4.56	4.79
14 Critically evaluate the educational efficacy of simulation	246	4.36	4.00	0.97	4.23	4.48
15 Demonstrate and analyse debriefing and feedback methods used in SBE	246	4.64	5.00	1.03	4.51	4.77
16 Design and facilitate a simulated learning event	245	4.30	4.00	1.12	4.16	4.44
17 Develop an evaluation plan of a simulated learning event	245	4.15	4.00	1.12	4.01	4.29
E1: Manikin-based simulation						
18 Describe the range of manikins available for healthcare simulations	130	4.92	5.00	0.89	4.76	5.08
19 Identify features of manikins (e.g. mobility, immediacy of feedback etc.)	130	4.78	5.00	0.93	4.62	4.95
20 Demonstrate how to decides which manikin to use	132	4.57	5.00	1.03	4.38	4.75
21 Describe the benefits and difficulties of programming a manikin	130	4.43	5.00	1.19	4.22	4.64
22 Plan and demonstrate a manikin-based simulation activity	125	4.63	5.00	1.08	4.44	4.82
E2: Simulated patient (SP) methodology						
23 Describe contemporary applications of SP methodology	69	4.94	5.00	0.79	4.75	5.13
24 Discuss responsibilities of SPs in teaching sessions	69	5.00	5.00	0.91	4.78	5.22
25 Create SP roles for teaching and assessment	69	4.78	5.00	1.13	4.51	5.05
26 Describe a systematic approach to SP training for role portrayal	69	5.06	5.00	0.84	4.85	5.26
27 Consider the content of feedback from SPs to trainees	69	4.97	5.00	0.95	4.74	5.20
28 Describe principles of feedback as they apply to SP-based education	69	4.93	5.00	0.94	4.70	5.15
29 Describe the practicalities of training SPs and SP educators (including assessment)	68	4.82	5.00	1.04	4.57	5.07
E3: Virtual environments						
30 Describe the advantages and disadvantages of virtual environments and their applications	45	4.43	4.00	1.00	4.13	4.74
31 Describe a range of virtual environments	45	4.75	5.00	1.06	4.43	5.07
32 Articulate the role of educational design in effective virtual environments	44	4.39	4.00	1.02	4.08	4.70
33 Describe issues in developing virtual environments	45	4.41	4.00	1.06	4.09	4.73
34 Teach simulation educators to review virtual environments for SBE	45	4.25	4.00	1.14	3.90	4.60
E4: Hybrid simulation (Patient focused simulation)						
35 Outline key elements of SP-based methodology	144	4.71	5.00	0.95	4.55	4.86
36 Describe the rationale for hybrid simulations	144	4.90	5.00	0.92	4.74	5.05
37 Define patient-focused simulations	144	4.84	5.00	0.95	4.68	5.00
38 Outline the scope practice of hybrid simulations	144	4.76	5.00	0.92	4.60	4.91
39 Describe relevant theory underpinning patient-focused simulations	144	4.67	5.00	1.02	4.50	4.84
40 Review written scenarios for patient-focused simulation	144	4.50	5.00	1.07	4.32	4.67
41 Describe a systematic approach to SP training for role portrayal	144	4.87	5.00	0.99	4.70	5.03
42 Consider the role of feedback in patient-focused simulations	143	5.00	5.00	0.87	4.86	5.14
43 Practice training SPs for feedback in patient-focused simulations	143	4.62	5.00	1.20	4.42	4.82
44 Describe the practicalities of training SPs and SP educators in patient-focused simulations (including assessment)	143	4.62	5.00	1.05	4.44	4.79
T1: Equipment sustainability and task trainers						
45 Describe the roles of the simulation specialist	59	4.83	5.00	0.86	4.58	5.08
46 Demonstrate ability to perform hazard analysis and risk assessment for simulation activities	59	4.81	5.00	0.89	4.55	5.07
47 Describe the value of preventative maintenance and how it can be accomplished	59	4.90	5.00	0.72	4.69	5.11
48 Describe the concept of fidelity in relation to this role	59	5.13	5.00	0.67	4.93	5.32
49 Discuss the needs of actors involved in simulation	59	4.88	5.00	0.87	4.62	5.13
T2: Basic manikins and moulage						
50 Articulate the capabilities and limitations of various manikins	55	4.59	5.00	1.02	4.31	4.87
51 Identify various cables and connections and what they do	55	4.41	5.00	1.35	4.04	4.78
52 Describe the basics of programming manikins	55	4.17	5.00	1.34	3.80	4.53
53 Familiarization with manikin parts and systems	54	4.57	5.00	1.14	4.26	4.89
T3: Advanced manikins and audio visual (AV) systems						
54 Review the different types of AV systems	51	4.33	4.00	1.07	4.02	4.63
55 Review the different AV connectors	50	4.37	5.00	1.24	4.01	4.72
T4: Delivery of scenarios						
56 Articulate the process required to the design and delivery of a purposeful scenario	54	4.31	5.00	1.24	3.98	4.65
57 Demonstrate an ability to develop, prepare and deliver an effective scenario	54	4.22	5.00	1.28	3.87	4.57
58 Use reflection and constructive feedback to critique	54	4.33	5.00	1.13	4.02	4.64

HWA’s original proposal was that AusSETT alumni would support the training of 6000 further educators and technicians through a separately funded program. National train-the-trainer programs are rare in this field and we have not identified any national programs for simulation educators and simulator technicians on a global scale. This paper describes the AusSETT Program and the associated objectives-oriented program evaluation.

The institutional members, responsible for developing the AusSETT Program, came from four states and included leading simulation education groups. The development team was comprised of ten authors with clinical (e.g. medicine, nursing, paramedicine, operating theatre technician), content (e.g. human factors, communication, assessment) and process (e.g. education, simulation modality) backgrounds. Authors have experience of several simulation modalities including manikins, task trainers, hybrid simulation, simulated patients, confederates, virtual patients and virtual environments and in different settings such as purpose-built centres, non-specialist settings and in situ. They have between six and 35 years experience of using simulation as an educational method to support learning in healthcare. All authors lived and worked in Australia at the time of development. The ten reviewers had a similar profile although three were from Canada and the United Kingdom.

The development team designed a curriculum with three key features. First, the Program emphasised educational principles based on published healthcare simulation literature. Second, the Program included a broad coverage of simulation modalities including manikins, task trainers, simulated patients and virtual environments. Third, the Program comprised both e-learning and workshops. This approach ensured that the Program was accessible and applicable to diverse simulated learning environments. There were two shared core modules for educators and technicians (C1 and C2). Educators and technicians each completed four additional modules (E1 to E4 and T1 to T4 respectively). Each module was designed to take between four and eight hours. Table 2 summarises the module content and format. Overall goals of the Program were to enable participants to:

**Table 2:** Brief description of AusSETT Program modules

Module number	Module name	Module description	Format
C1	Simulation-based education: Contemporary issues for healthcare professions	This module covers the rationale for simulation-based education (SBE) drawing on practices from high-reliability industries. Theories are explored as they relate to different simulation modalities (e.g. manikins, task trainers, simulated patients, virtual patients, gaming etc.) and domains (e.g. knowledge, attitudes and skills). The relevance of simulation to safety and quality in healthcare are highlighted. Participants consider historical and contemporary approaches to SBE with attention to the benefits and challenges in health professions education. Evidence for SBE is presented. The module offers key content to ensure that all participants have a common understanding of basic principles for SBE. The module is essential prior to attending any workshops.	e-learning
C2	Training simulation educators	This module covers basic principles of “train-the-trainer” programs, as well as the opportunity to practice and/or learn new simulation skills. One of the educator’s roles is to provide a safe learning environment, especially during feedback and debriefing. The module also explores how to teach and evaluate debriefing skills. Reference is made to the range of debriefing approaches used in different centres across Australia. Because there is no high level evidence for the use of a single debriefing approach, the Program is distinguished by its flexibility and willingness to support multiple approaches. This is a core module for all simulation educators and technicians.	e-learning; workshop
E1	Manikin-based simulation	This module covers the core aspects of manikin and task trainer simulations. This includes an overview of different manikin and task trainer capabilities and the impact these have on SBE. Scenario development, basic programming and scenario delivery are explored together with the concept of fit for purpose manikins.	e-learning; workshop
E2	Simulated patient (SP) methodology	In this module, participants explore fundamentals of SP practices including recruitment, selection, training for role portrayal and the role of SPs in feedback. The breadth of SP practices is explored and potential opportunities for applications across different contexts.	e-learning; workshop
E3	Virtual environments	This module enables participants to explore virtual environments as an educational method. Formal models and different design modalities provide participants with frameworks for practice. This module also provides an opportunity for participants to debate key issues in virtual patient methodologies.	e-learning
E4	Hybrid simulation (Patient focused simulation)	In this module, participants focus on blending simulation modalities. Examples include the linking of SPs with task trainers (patient focused simulation) to support the integration of all skills necessary for safe and effective clinical performance. Participants explore scenario development, briefing and feedback/debriefing.	e-learning; workshop
T1	Equipment sustainability and task trainers	This module focuses on the principles of equipment use, repair and maintenance and the role of technicians in supporting learning.	e-learning; workshop
T2	Basic manikins and moulage	This module focuses on the technical elements of the operation, troubleshooting and maintenance requirements of “low technology” manikins (e.g. Megacode Kelly and Megacode Kid). Participants develop skills in the operations and functionality of the manikins, including their limitations. Moulage techniques are explored including the use of realistic cuts, grazes and bruises and full body multi-trauma and burns.	e-learning; workshop
T3	Advanced manikins and audio visual (AV) systems	This module is designed for technicians who work with advanced manikins (e.g. SimMan, SimBaby, iStan, Meti Man) and AV systems. The module focuses on the set-up, operation, troubleshooting and maintenance requirements of manikins and AV systems, including their functionality and limitations.	e-learning; workshop
T4	Delivery of scenarios	This module T1, T2 and T3 enabling participants to develop, set-up and deliver a scenario. Participants are expected to set up the AV system, manikins and environment and deliver the scenario, with peers. The module emphasizes the importance of cooperative efforts of educators and technicians in all phases of SBE.	e-learning; workshop

Table 2 shows the topics covered in the AusSETT Program and there were a total of 10 modules. The first column lists the numbering code for each subject where ‘C’ denotes core, while ‘E’ and ‘T’ refer to education and technician module respectively. The second column details the full title of the topics covered. The third column describes what was covered within each module. The fourth column clarify the delivery method of each subject; either via e-learning platform or workshop

- Apply knowledge and skills in the design and delivery of SBE; and
- Practice contemporary training approaches that influence, motivate and inspire learners.

The Program combined e-learning and workshops and was free to participants. HWA networks nominated participants. The learning management system was custom designed while the e-portfolio was a proprietary product (PebblePad) [9]. Workshops were conducted across the country with six to 28 participants. Over ten months, 230 educators completed the Program.

In this paper we report the evaluation addressing the following questions:

- To what extent can a national program for healthcare simulation educators and technicians prepare participants to support others in using SBE?
- What are the strengths and limitations of the Program as identified in structure, process and outcome factors?

## Methods

We adopted an objectives-oriented program evaluation approach [10] exploring the structure, process and outcome [11]. In the context of the AusSETT Program examples of *structure* include the range of skills and abilities of the team to develop and deliver the Program, the sequence and validity of the content (including the learning objectives), the scope of the Program within the time frame and the physical infrastructure for delivery. Examples of *process* include the educational methods such as the e-learning, workshop format, the e-portfolio and feedback mechanisms etc. *Outcome* refers to the extent to which the program goals were met. These were mainly focused on outcomes for participants and these were made clear in the learning objectives (Table 1). However, there were likely to be unexpected outcomes too. It was beyond the scope of our evaluation to monitor these changes but we anticipated identifying some through qualitative interviews with participants.

Data was collected using a range of methods, and are described below. Instruments are summarised in Table 3 and examples provided in Additional file 1.

**Table 3:** Summary of instruments used in the evaluation of the AusSETT Program

	Instrument	Respondent	Evaluation component	Example
1	Module review	Consortium experts External experts	Structure Process	Not applicable
2	Baseline questionnaires	Participants Faculty	Structure	Additional file 1
3	Observation of workshops	External experts	Structure Process Outcomes	Additional file 1
4	End of module evaluations	Participants	Process Outcomes	Additional file 1
5	Individual interviews	Participants	Structure ProcessOutcomes	Additional file 1

Data in the study were obtained via 5 instruments presented in Table 3. These are itemised in the first column and include module reviews, baseline questionnaires, workshop observations, end of module evaluations as well as individual interviews. The second column in the box specifies who were involved during the use of each evaluation instrument. The third column indicates which aspect of the program was being evaluated when each instrument was applied; structure, process and outcomes. The last column point to an example of each instrument within the Additional file 1

### Module review

Program partners and external subject matter experts reviewed each module prior to delivery to ensure content was contemporary, accurate and contextualised. External reviewers were identified through collegial networks and published literature. Written feedback was sought and used to adjust content and methods. Internal review was undertaken throughout the development and implementation process – at meetings, in response to specific requests for feedback, and at workshops. Although all modules were sent externally for review with the same instruction, not all received formal written responses. Authors who nominated subject matter experts and wrote personally to the reviewers received detailed written feedback. Feedback was iteratively incorporated into modules and text of feedback was thematically analysed.

### Baseline questionnaires

Online questionnaires were completed by all participants and faculty before commencing the Program and sought demographic and professional characteristics, and current practices of SBE (Additional file 1). Data were analysed using descriptive statistics in SPSS and content analysis.

### Observation of workshops

In each state and territory, an external observer was invited to observe workshops (Additional file 1). The eighteen observers were selected based on their expertise in health professional education, simulation education and higher education. Observers used a template for each workshop enabling consistency. Free text comments were collated and content analysis was used to identify commonly recurring themes. They were not trained to use the semi-structured form relying on their expert judgement.

### End of module evaluations

On completion of each e-learning module, participants were asked to rate the degree to which they met learning objectives and the value of the educational methods in attaining them. Prior to the program, we aspired for mean scores to equal or exceed 4.5 on the 6-point rating scale (1 = *not at all* met to 6 = *completely* met). That is, participants have more than ‘moderate’ achievement of learning objectives. Participants were also asked to identify five things they learned during the module that would inform their ability to train others, what worked well in the module and what needed to be improved (Additional file 1). Data was analysed using descriptive statistics. Conventional content analysis was used to collate free text comments [12].

### Individual interviews

The goal of the individual telephone interviews was to gain deeper insight into participants’ experiences. Interviews were also used to interrogate other evaluation data (Additional file 1). Purposeful sampling identified participants across jurisdictions, health professional group and experience in simulation-based education. A topic guide was developed with overall Program goals in mind and to explore ‘unexpected’ outcomes. Telephone interviews were conducted by an independent researcher employed for the task and were scheduled for up to one hour. They were recorded and transcribed prior to thematic analysis [13]. JH and DN undertook the thematic analysis using an inductive approach. Key themes were identified independently and then negotiated. The data was then checked again for confirming and disconfirming evidence. An audit trail of analysis was maintained. The interviews were carried out two months after the completion of the Program.

Numerical data was analysed by JH while both JH and DN undertook the analysis of textual data. The Monash University Human Research Ethics Committee approved the study (#CF12/0244-2012000099). Written consent for participation was obtained from the participants to include their data in this study.

## Results

### Module review

Recommendations included raising the profile of interprofessional SBE, distinguishing essential from recommended or optional readings, indicating areas of overlap between modules, providing a glossary of terms and checking that content addressed general rather than specific principles of SBE. These structural concerns were undertaken and addressed.

### Baseline questionnaire

The demographic data of the participants are presented in Table 4. Sixty-three percent (*n* = 172) of the participants were female and 37 % (*n* = 103) were male while the remainder of participants did not respond to this item. Percentages are calculated on those reporting for each item and the numbers presented in brackets to aid understanding. Thirty-seven percent (*n* = 100) were 40 years of age or less and 63 % (*n* = 168) were 41 years of age or older. Seventy eight percent (*n* = 235) of participants chose to complete the educator stream and 22 % (*n* = 65) opted for the technician stream. Fifty-five percent (*n* = 153) were employed by public health care service, 29 % (*n* = 80) and 8 % (*n* = 23) were employed by universities and private health care services, respectively. The remainder were employed by Technical and Further Education (TAFE) Colleges (4 %, *n* = 10) and other organisations (4 %, *n* = 11). Over half of the participants (52 %, *n* = 133) taught both undergraduate and postgraduate students, while 28 % (*n* = 73) only taught at postgraduate level. Twenty percent (*n* = 51) taught only undergraduates and the balance did not respond.

**Table 4:** Participants demographic information

	Number of participants	Percentage
Gender		
Female	172	63
Male	103	37
Age group		
40 years of age or less	100	37
41 years of age or older	168	63
Choice of stream		
Educator	235	78
Technician	65	22
Principle employer		
Public health care service	153	55
University	80	29
Private health care service	23	8
Vocational education sector	10	4
Other	11	4
Target group for teaching		
Undergraduate & postgraduate	133	52
Postgraduate only	73	28
Undergraduate only	51	20
Current role		
Nursing/midwifery	161	57
Medical	49	17
Allied health	34	12
Educational/administrative or other non-clinical	37	13
Simulation experience		
Spent 10 h of less per month	135	53
Spent over 80 h per month	18	7
Had previous simulation training	102	40

The majority of participants had a nursing or midwifery background (57 %, *n* = 161), 17 % (*n* = 49) had a medical background and 12 % (*n* = 34) were from allied health with fourteen professions noted, including physiotherapy, paramedicine and occupational therapy. Thirteen percent (*n* = 37) described educational, administrative or other non-clinical backgrounds.

On simulation experience, the majority of participants (53 %, *n* = 135) spent ten hours or less per month on simulation activities, including 25 participants with no simulation experience. Seven percent (*n* = 18) indicated they spent over 80 h per month on simulation activities, which would indicate a full-time simulation education position. Forty percent (*n* = 102) of participants had previous simulation training, such as a debriefing course, an Advance Life Support instructor course, train-the-trainer courses or fellowships. Of these, nineteen had undertaken or were undertaking post-graduate studies in simulation.

Table 5 provides a content analysis of free text on the participants’ views on simulation and the AusSETT Program. The ‘top five’ themes are provided. This analysis indicates that skills such as debriefing or learner engagement are the areas of most challenge for participants; manikin-based simulation is the most common type of simulation modality used by the participants; and 61 % of participants enrolled in the AusSETT Program to improve their own skills, knowledge or understanding with 19 % enrolling to train others. Additionally, they appreciated that the Program was free, that they were nominated to attend and that they had dedicated time to spend in professional development.

**Table 5:** Content analysis of participants’ views on simulation and the AusSETT Program

	Number
Top five themes: Most challenging aspect of simulation-based education	259
1 Debriefing or aspects of debriefing such as feedback	44
2 Issuesof authenticity, such as ‘making it real’	43
3 Engagement of staff or students as a challenge	40
4 Finding the ‘time’ to conduct simulation-based education	27
5 Resources, such as money or equipment	21
Top five themes: Areas of interest in simulation practice	262
1 Debriefing or aspects of debriefing such as feedback	43
2 Manikin modality or simulation centre type simulation	28
3 Professional skills, with 15 specifically mentioning teamwork	28
4 Scenario development	25
5 All aspects of simulation	21
Top five themes: Reasons for enrolling in the AusSETT Program	270
1 To develop, expand or consolidate knowledge and skills	164
2 To train/support others	52
3 A passion, interest or belief in simulation-based education	40
4 Invited, nominated or part of employment	40
5 Directly relevant to employment role	25

### Observation of workshops

The key outcomes of observer reports suggested that educational methods supported participants in meeting learning objectives. That is, there was alignment between content and process and perceived participants’ needs. Observers noted effective management of group dynamics, the creation of safety for participants and the benefits of learning across consecutive days with relationships forming between participants and faculty. Recommendations included making better use of e-learning in workshop activities, a similar template for all slides, extended time for discussion of local issues, and longer review time for some experiential activities.

### End of module evaluations

Table 1 summarises the participants’ evaluation of the attainment of learning objectives. There were 58 learning objectives in total with 17 in the core modules, 27 in the educator modules and 14 in the technician modules. Overall, 37 learning objectives exceeded 4.5 while the remaining 21 learning objectives were in the range of 4.15 to 4.43. That is, overall participants reported at least *partially* meeting most objectives. For educator modules, E2 (7 objectives) and E4 (10 objectives) all learning objectives exceeded 4.5. For technician modules, all objectives in T1 exceeded 4.5 while in T2, two of four objectives exceeded 4.5 while neither T3 nor T4 reached this standard. Within the core modules (C1 and C2), one of the highest rated learning objectives was to *describe various types of simulation activities* with a mean of 4.72 (Median = 5.00, SD = 0.95, 95 % confidence interval = 4.60 to 4.84). The lowest rated learning objective was to *develop an evaluation plan of a simulated learning event* with a mean of 4.15 (Median = 4.00, SD = 1.12, 95 % confidence interval = 4.01 to 4.29).

A mean of 5.06 (Median = 5.00, SD = 0.84, 95 % confidence interval = 4.85 to 5.26) was reported for *describe a systematic approach to simulated patient (SP) training for role portrayal*. This is the highest reported learning objective within the educator stream. The lowest reported learning objective within the educator stream was for *teach simulation educators to review virtual environments for SBE* (Mean = 4.25, Median = 4.00, SD = 1.14, 95 % confidence interval = 3.90 to 4.60). In the technician stream, the highest rated learning objective was for *describe the concept of fidelity in relation to [specialist/technician] role* with a mean of 5.13 (Median = 5.00, SD = 0.67, 95 % confidence interval = 4.93 to 5.32). The lowest rated learning objective was for *describe the basics of programming manikins* (Mean = 4.17, Median = 5.00, SD = 1.14, 95 % confidence interval = 4.26 to 4.89).

### Content analysis

Qualitative responses for module evaluation covered three areas: i) what worked well; ii) what needs improvement; and iii) things that participants had learned. As the data collected provided a large body of information, only selected responses are included.

### What worked well

There were many successes with the modules such as the “open” small and large group discussions, high quality facilitation, the opportunity to learn from each other, debriefing exercises especially with observing different styles of debriefing, the use of non-clinical scenarios for learning about SBE principles, e-learning exercises and debriefing resources, the reference to educational theory relevant to SBE and the breadth of simulation modalities.

These successes are illustrated by examples of text from a core module (C2):

*“It was a great discussion-based learning experience, drawing on others’ knowledge and experiences to create themes around simulation and debriefing; The faculty were extremely supportive”*

*“Different simulation modalities and the understanding that you don’t need the best simulator to achieve the outcome”*

*“I enjoyed the hands on part of the module where we designed a simulation exercise and needed to give feedback; Also the chance to meet other people trying to do the same thing or with other ideas/experience was great; As I am very new to this area it was all a bit daunting, so hearing some of their tips/techniques was helpful”*

### What can be improved

The need to improve the learning management system, the use of the e-portfolio and more time for completing the preparation prior to attending workshops were raised by the participants.

*“The response boxes need to allow for editing. After brainstorming some ideas and submitting I realised that I could not re-enter to finalise my ideas; Scrolling between 2 pages to re-read quotes then meant that what I had entered into the text box had been lost; Quite frustrating…” (C1)*

*“The computer interface; Many of my responses were partially or fully deleted on submission; I was unable to then edit the response after submission and 90 % of my scenario was not loaded; I found this extremely frustrating and do not feel the saved answers reflect well either what I actually wrote or my effort in this module.” (E1)*

*“Couldn’t access the pebblepad” (C2)*

*“I have had difficulties accessing many of the links and the audio component, so that made it difficult; There was also a huge amount of readings required making it very lengthy to work through” (E2)*

Some participants also expressed the need for information about when the next phase of the Program would commence.

*“I think AusSETT needs to have a clearer idea of where this project is going, as the presenters just didn’t have the answers about the next few steps; I worry that by the time the project is developed fully, I will have forgotten what I am supposed to train others in.” (C2)*

### Content analysis of participants’ reported learning

Participants were requested to list up to five things they had learned during each module. This provided further insight to whether the objectives of the sessions were met. Only responses from a core module (C2) are presented as the data collected yielded a large amount of information. Some of these statements covered one or more of the successes reported above. The top five results – practical SBE skills, reinforcement of important processes in SBE, access to resources for SBE, theories that inform SBE and generic teaching approaches - suggest that the module offered consolidation and/or development of knowledge and skills (Table 6). The module also provided helpful resources for the participants.

**Table 6:** Content analysis of top five things learned from Module C2: Training simulation educators (*n* = 102)

		Number
1	Practical skills (e.g. using workshop slides, giving feedback, debriefing etc.)	55
2	Reinforced important processes of SBE (e.g. learning objectives, testing scenarios, debriefing practices etc.)	25
3	Obtained resources/tools/template (e.g. access to resources)	18
4	Theoretical knowledge (e.g. theories that inform simulation practice)	14
5	Processes that worked well (e.g. managing group dynamics, interactive workshops, different levels of expertise, co-facilitation etc.)	6

### Individual interviews

Nine interviews were carried out between August and November 2012. Each interview took between 18 to 30 min with 239 min of data obtained. Feedback from the interview triangulated the end of the module evaluation data. Overall, positive general thoughts and feelings for the Program were reported and reinforced the earlier data. The interviews identified an overarching theme: the impact of the Program. Three specific areas of impact were noted; a) personal; b) organisational; and c) professional community. Verbatim statements from the interview data are presented in Table 7. Participants’ reported that the Program had boosted their confidence as a trainer of SBE. The Program reinforced as well as expanded their knowledge on SBE, increasing their interest in other similar programs for furthering their professional development. These individuals reported that they became “champions” for the use of SBE within their organisations. Some of them reported that their organisations had taken an active interest in SBE as a direct consequence of the AusSETT Program. The Program also encouraged inter-professional learning within their organisations. Development of a community of practice was also an outcome as some participants reported that they had kept in contact with others to exchange ideas and information on SBE, interacting regularly and that AusSETT Program offered a shared language as well as repository of resources.

**Table 7:** Verbatim statements from interviews with participants with key themes

Personal impact	
1. Builds self-confidence	
I went in as a person involved in simulation for about four years and stepping out of it I felt that, even with my meagre period of experiences in this field, I stepped up feeling more confident, I stepped out knowing that I had specific things that I could contribute to the running of a simulation centre and the use of simulation in education. (Participant 1)	
I do the tech programme, the university simulation sessions, which are repeated every year. That sort of the length and breadth of what I do, but I’m stopping in the next six months looking perhaps doing some things with local GPs which would be taking simulation to them in their rooms to do ALS training. And that’s probably grown out of some confidence that I got from the AusSETT Program, and the concept of trying to spread simulation. (Participant 8)	
2. Reinforce knowledge on simulation	
I think it’s reinforced the usefulness of simulation (Participant 2)	
I think it’s reinforced a few things, it’s reinforced the merits of simulation in particular areas where there has been some research, it’s reinforced my view that we still need to do a fair bit of work around researching what constitutes effective simulation training and it has reinforced something for me…. (Participant 6)	
3. Gained new knowledge on simulation	
… see how other people do things, and then you learn that there are different ways of doing things, and I think that probably enriches you as an individual by having a look at a little bit of everything. (Participant 4)	
it’s certainly influenced my thinking and certainly influenced my knowledge in seeing what’s out there and what other people are doing and how it’s being used in other settings. (Participant 9)	
4. Interest in other simulation program for professional development	
I’m actually looking at doing a formal course now, as like the follow-on, perhaps a [removed] course or something like that. It’s certainly got my interest and I’m really keen to do more. So it’s been successful in that regard. (Participant 5)	
I think my interest from here on is obviously to get a sense of the next steps and getting a clearer picture on who have the intention of doing further training and then starting to bring that together, because I think we may get some useful feedback with collaboration with others who wish to be facilitators. (Participant 6)	
Organisational impact	
1. Championing the use of simulation within organisation	
… its allowed a broader range of people to start thinking about it and getting more knowledge about it, and then hopefully discussing it more in their workplace. (Participant 2)	
I guess just opportunities to look at sharing some of what I’ve got from the program with others in our discipline as well who I know are really keen or get them along to the program to hear it as well. (Participant 5)	
2. Use of simulation	
We very much do a lot of mannequin training, and she very much opened up the doors to this hybrid system and using real people…I think that’s very interesting, and we actually have done a little bit now and we’ve had good feedback. (Participant 4)	
3. Encouraging inter-professional learning	
I’m moving simulations so one of the people in my group for this semester for example is from dentistry and veterinary science so we’re helping her set up some simulation in dentistry and also we’ve spoken about it with veterinary science as well. So we’re sort of looking at expanding it… (Participant 3)	
Professional community impact	
I think it has the potential to open people’s eyes to, that there is community out there and you can get support, you can get training, and you can go somewhere and find information or ask someone for information. So I guess that’s going back to that community sort of aspect there. (Participant 7)	
I think we developed a quite cohesive group for the three days we were there and I know that we have, some of us have met up again at conferences and we’re happy, because we exchange phone numbers and emails etc.…. So I think that, yes, it was very good at developing, networking and collegial relationships. (Participant 3)	
I do the tech programme, the university simulation sessions, which are repeated every year. That sort of the length and breadth of what I do, but I’m stopping in the next six months looking perhaps doing some things with local GPs which would be taking simulation to them in their rooms to do ALS training. And that’s probably grown out of some confidence that I got from the AusSETT Program, and the concept of trying to spread simulation. (Participant 8)	

## Discussion

The results support the notion that the AusSETT Program was an important national initiative. The interviews and observations indicate the value that participants place upon the program and how access to the Program has resulted in significant goodwill in the healthcare simulation professional community. The overwhelming sense was that participants had high standards, were aspirational for their practices and wanted to make a difference using simulation as an educational method. However, the impact of the Program cannot be fully measured until the participants themselves offer simulation educator and technician training.

### Structural factors

Many strengths of the Program were primarily structural. They included the building of a significant and enduring repository of contemporary resources (e-learning and workshops), and its offering in urban and rural regions, across jurisdictions, simulation modalities and professions. The Program also had a national profile with the representativeness of the development team. The module review process enhanced the strength of the structural factors.

Enabling structural factors from participants’ perspectives included being identified to attend the Program, some dedicated time off work to focus on professional development, networking associated with spending time immersed in SBE and offering the Program free of charge to participants. Other enablers included the access to e-learning. In fact, the e-learning also formed a significant structural constraint, particularly with respect to the functionality of features. Additionally, participants largely resisted the use of PebblePad, which did not seem related to the tool itself but to its use in this particular context. At a higher level, a constraining factor was that, at the time of the Program delivery, the next steps were not known to the Program faculty. Although HWA had longer-term goals, the mechanisms of government, especially in relation to budgets, did not permit sharing of plans in a more timely fashion. This created a barrier for engagement by individuals and institutions with the Program.

### Process factors

Participants and workshop observers identified the types of active learning opportunities as a strength of the Program. Indeed, participants identified a key point of learning as including a series of ‘processes that worked well’, which were modelled during the process factors. The need to have skilled facilitators was reinforced by workshop observers; this may be a key enabling process factor for any future Program.

### Outcome factors

Participants’ reported that they largely met learning objectives. Where objectives were not met as well as our pre-set standard, this may have related to the relative inexperience of some participants. Although observers reported alignment of content with perceived needs of participants, this may not have been achieved in every workshop as not all were observed.

It is worth noting is that there was no assessment associated with the AusSETT Program so we have no way of knowing if participants did achieve competence relevant to the learning objectives. This may be seen as both an enabling factor – in terms of access - and a constraining factor – in terms of accreditation – depending on perspective. Our philosophical view was that we wanted to provide continuous professional development and that at this nascent stage of development of a national community of practice of healthcare simulation educators and technicians, assessment may not have been well received. Also, within the funding model it was not possible to offer robust assessment, but this may be a future step.

Overall, the evaluation results give some indication that a national program for simulation educators and technicians is valuable for supporting others in using simulation as an educational method, at least from the perspectives of those who participated as future ‘trainers’. There is one major caveat. Many participants had no or limited experience with simulation and very few had formal qualifications. Indeed the first reason given for enrolling with the program was to “develop, expand or consolidate” skills. The AusSETT program was intended for experienced simulation experts, who could support others, not for those who were novices. In many ways this suggests that a ‘training’ program rather than a ‘train-the-trainer’ (‘educate-the-educator’) program may be more aligned with participants’ needs. However, the question of training others will only be fully answered once the AusSETT alumni have had an opportunity to offer professional development to other cohorts. However, there is no question that the Program promoted individual development and ignited interest in SBE in local facilities.

The contractual agreement strongly influenced some elements of the Program’s development and implementation and some of the language used to describe simulation practices – such as those of educator and technician. Although the opportunity to pilot the Program is likely to have reduced the scale of iterative development, the actual adjustments to the Program were minimal. The funding body requested a train-the-trainer model but in many respects we adopted a perspective of ‘educate-the-educator’, especially with respect to sharing theoretical underpinnings of many facets of simulation-based education.

### Strengths and limitations of the study

The strengths of the study were the objectives-oriented program evaluation design, mixed methods, multiple sources of data (participants and subject matter experts) and the multiple data points collecting baseline, immediate responses after e-learning and workshops and then those in the longer term (two-months), at least for some participants.

Limitations of the study are that not all participants gave permission to publish their data which accounts for some of the discrepancies in reporting. The data is self-report which may not reflect the actual practices of participants. As internal evaluators, our own biases and assumptions may have influenced the data analysis and that which we have chosen to share from the very large data set. However, we employed an *independent* researcher (JH) to play a leading role in data analysis. Finally, in the absence of data-driven accounts of similar programs, it is not possible to discuss our findings in that context.

## Conclusions

In summary, the evaluation of a national ‘train-the-trainer’ program for simulation educators and technicians suggested the value and need for a nationwide approach to training educators in simulation methodologies. The multi-layered objectives-oriented evaluation identified strengths and weaknesses of the Program with specific recommendations for improving its’ content and processes. We hope that sharing the details of learning objectives and our broader experiences may inform others embarking on simulation educator and technician training course development. The ‘national’ identity was achieved through the scale and diversity of the program in terms of simulation modalities, the geographical spread, breadth of professional disciplines, range of educational methods and the multi-institutional development.

## Additional file

Additional file 1: Baseline questionnaires on participants’ views of simulation and the AusSETT Program. (PDF 45 kb)
