# Supplementary material for: A national training program for simulation educators and technicians: evaluation strategy and outcomes
Source: BMC Med Educ. 2016 Jan 22;16:25. doi: 10.1186/s12909-016-0548-x (PMC4722779; doi:10.1186/s12909-016-0548-x)
Supplement: Additional file 1: — Baseline questionnaires on participants’ views of simulation and the AusSETT Program. (PDF 45 kb) [file 12909_2016_548_MOESM1_ESM.pdf]

Additional file 1

Baseline questionnaires on participants' views of simulation and the AusSETT Program

We would like to survey you before and after the AusSETT Program. This is to evaluate the AusSETT Program and provide feedback to Health Workforce Australia. This is just one thread of the evaluation strategy and we appreciate your involvement. The questions are designed to address the goals of the AusSETT Program. We also seek information on your personal and professional characteristics. All personal and professional information will be collated. Individual qualitative responses will be de-identified.

Qualifications and primary healthcare discipline

1. What is your primary healthcare discipline? Options provided and other category
2. What year did you graduate in your primary healthcare discipline (or nominated other)? Four digit number response
3. Which institution awarded your primary healthcare discipline? [e.g. Gippsland TAFE, University of Queensland etc]. Free text space for response
4. Please list all your formal qualifications and memberships [e.g. BSc (Nurs), RN, GCHPE, etc] Free text space for response
5. What is your healthcare discipline specialty? [e.g. acute care, care fo the elderly, palliative care etc) Free text
6. Who is your principle employer? List options as per question 1 e.g. Public health care service, Private health care service, University, TAFE, other…, please specify
7. Have you undertaken formal study/training in simulation-based education? Yes/No
    a. If yes, outline the nature of the study/training. (< 200 words)
    b. Who was the provider/s of the training? (<200 words)
    c. Overall, to what extent was the training useful? (<200 words)

Current professional practice

8. Give a brief overview of your current clinical responsibilities? (<200 words)
9. Give a brief overview of your current teaching responsibilities (not simulation-based)? (<200 words)
10. Please describe your students/participants. (e.g. undergraduate nurses years 1-3; postgraduate; interprofessional including doctors, nurses, allied health professionals etc)(<200 words)
11. Give a brief overview of your current simulation-based teaching responsibilities? (<200 words)
12. Approximately how many hours each *month* do you spend in simulation-based education? None, 0-10, 11-20, 21-30, 31-40, 41-50, 51-60, 61-70, 71-80, >80 drop down menu
13. Describe the role, if any, of educational theory within your teaching practice. (<200 words)
14. What do you find most challenging in simulation-based teaching and learning? (<200 words)
15. What aspects of simulation-based education are you most interested in? (<200 words)
16. Can you please outline why you have enrolled in the AusSETT Program? (<200 words)
17. What are your expectations of the AusSETT Program? (<200 words)

18. What benefits are you expecting from successful completion of the AusSETT Program? (<200 words)
19. Would you be interested in further simulation education training? Yes/No

Demographics

20. What is your year of birth? 4 digit space
21. What is your sex? Male/Female
22. What is your postcode? 4 digit space
23. What is your country of birth? Either a standard list or Australia, Other, please specify

Observation of workshops

Observations of the AusSETT Program

Please provide feedback on the following components of the workshop. There will be overlap between headings so feel free to report on topics under your preferred headings. We would like to include your report as an appendix in our final submission to Health Workforce Australia (HWA) on the AusSETT program. We will acknowledge you as one of the observers of AusSETT although your report will be anonymous.

Please complete a separate report for each module.

Module:
Date:
Start time:
Finish time:
Venue:
Facilitators: Names will not be included in the report
Number of participants:
Ambience:

1. Overall comments
2. Workshop content – *level for learners, amount, breadth and depth for time frame*
3. Workshop methods – *including facilitation, engagement of learners with activities, timing, transitions, instruction*
4. Participants – *diversity, levels of experience, interest, perceived attitudes, preparation*
5. Venue – *suitability, layout, flow, temperature, ease of access, technology, etc*
6. On-going use: *how do you think workshop would work in the future, with different participants, in different types of location.*
7. Recommendations

Example of module evaluations

To what extent did you meet the learning objectives?

	Not at all			Completely		
1. Describe contemporary applications of simulated patient (SP) methodology	1	2	3	4	5	6
2. Discuss responsibilities of SPs in teaching sessions	1	2	3	4	5	6
3. Create SP roles for teaching and assessment	1	2	3	4	5	6
4. Describe a systematic approach to SP training for role portrayal	1	2	3	4	5	6
5. Consider the content of feedback from SPs to trainees	1	2	3	4	5	6
6. Describe principles of feedback as they apply to SP-based education	1	2	3	4	5	6
7. Describe the practicalities of training SPs and SP educators (including assessment)	1	2	3	4	5	6

To what extent were the following methods helpful in meeting the learning objectives?

	Not at all			Completely		
1. Online exercise 1: Scope of SP practice	1	2	3	4	5	6
2. Online exercise 2: Writing SP-based scenarios	1	2	3	4	5	6
3. Online exercise 3: Training SPs for role portrayal	1	2	3	4	5	6
4. Online exercise 4: SPs and feedback	1	2	3	4	5	6
5. Online exercise 5: SPs in high stakes assessments	1	2	3	4	5	6
6. Workshop exercise 6: SP training - roles	1	2	3	4	5	6
7. Workshop exercise 7: Practicalities of SP-based education	1	2	3	4	5	6

What worked well in the module? (<250 words)

What needs improvement? (<250 words)

In your role as a trainer, list five things that you have learned in this module that will help you train SPs and SP educators.

1.

2.

3.

4.

5.

We recognize that new resources are emerging and that we may have overlooked some really important materials. Please make any recommendations for additional reference or resources.

Topic guide for individual interviews

1. Having completed the AusSETT Program, what are your thoughts and feelings about it?
2. Was there any stand out experience? If so, what was it? Why?
3. Specifically, what was helpful on the AusSETT Program?
4. Specifically, what was unhelpful on the AusSETT Program?
5. What are your thoughts and feelings about the online learning modules? What was helpful? What was unhelpful?
6. What are your thoughts and feelings about the portfolio? Was it helpful? Unhelpful? (PebblePad)… Do you document your practice using another type of portfolio or some other process…
7. What are your thoughts and feelings about the workshops? What was helpful? Unhelpful?
8. Was there content that you think we should have included that we did not?
9. What are your reactions to the workload of the AusSETT Program?
10. One of the goals of the AusSETT Program was to build a community of practice. To what extent did you feel part of a community of practice of simulation educators and technicians? Did AusSETT develop your community of practice? In what ways?
    a. Are you a member of the Australian Society for Simulation in Healthcare?
    b. Will you be attending SimHealth 2012?
11. What do you think will facilitate training others as simulation educators or technicians?
12. What do you think are the barriers for training others as simulation educators or technicians?
13. What are your views on the role of interprofessional simulation-based education?
14. Has the AusSETT Program influenced your thinking? If so, in what ways?
15. In what ways do you think standards in simulation-based education can best be established?
16. In what ways do you think standards in simulation-based education can best be maintained?
17. What do you believe are the most important elements of strategic planning in simulation-based education locally? Regionally? Nationally?
18. Is there anything else you would like to share with us about the AusSETT program?
